# Stanford Type B Aortic Dissection in a Twin Pregnancy With Marfan Syndrome: A Rare Obstetric Emergency—A Case Report

**DOI:** 10.1155/crog/4069281

**Published:** 2026-02-17

**Authors:** Oxana M. Zarudskaya, Alixandria F. Pfeiffer, Angela R. Boyd, John J. Byrne, Patrick Ramsey, Ildiko Agoston

**Affiliations:** ^1^ Division of Maternal-Fetal Medicine, Department of Obstetrics and Gynecology, University of Texas Health Science Center at San Antonio, San Antonio, Texas, USA, uthscsa.edu; ^2^ Division of Cardiology, University of Texas Health Science Center at San Antonio, San Antonio, Texas, USA, uthscsa.edu

**Keywords:** aortic dissection, case report, Marfan syndrome, pregnancy

## Abstract

The physiologic changes of pregnancy make pregnant women with Marfan syndrome (MFS) more vulnerable to complications of aortic disease. Significant hemodynamic changes in the third trimester and postpartum may explain the increased susceptibility to aortic complications during this time frame. The rate of cardiovascular complications in MFS is 2%–6%. We presented a case of Type B aortic dissection in a pregnant patient with MFS in the third trimester and normal evaluation at the initial prenatal assessment. This case report highlights the importance of comprehensive evaluation during pregnancy and the consideration of whole aorta imaging in addition to the routine transthoracic echocardiographic assessment of the aortic root.

## 1. Introduction

Marfan syndrome (MFS) is an autosomal dominant condition affecting approximately 1 in 3000 to 1 in 5000 individuals due to a gene encoding the connective tissue protein fibrillin‐1 (FBN‐1) mutation [1]. Affected individuals exhibit a wide range of phenotypic manifestations, affecting the skeletal, renal, ocular, skin, and cardiovascular organ systems.

The physiologic changes of pregnancy make pregnant women with MFS more vulnerable to complications of aortic disease. Hormonally mediated alterations in connective tissue and increased cardiac output may cause weakness of the arterial wall. Significant hemodynamic changes during the third and postpartum periods can lead to shear forces on the wall [2, 3]. This may explain the increased rate of aortic complications during this time frame in pregnancy. A literature review that included 1142 pregnant patients with MFS demonstrated that the rate of cardiovascular complications (primarily aortic complications and aortic dissection) was 2%–6% [2, 4].

The risk of dissection has been reported to be less than 1% if the aortic root diameter is less than 40 mm and greater than 10% if it exceeds 40 mm [4]. American College Of Cardiology Foundation (ACCF/AHA) thoracic aortic disease guidelines recommend against pregnancy in women with MFS and an aortic root > 40 mm or for prophylactic repair if they would like to become pregnant. It is important to note that women with MFS after prophylactic aortic root repair remain at risk for distal to the graft aortic dissection and may have a higher risk for type B dissection based on some smaller cohorts [5, 6].

The 2025 European Society of Cardiology on diagnosis and treatment of aortic disease and the 2014 Canadian Cardiovascular Society position statement on the management of thoracic aortic disease recommend against pregnancy when the aortic root is > 45 mm, or 41–45 mm in cases of a rapidly enlarging aortic root or family history of dissection/sudden death [4, 7].

We describe a case of Stanford Type B aortic dissection in a pregnant woman with twin gestation during the third trimester with MFS despite normal echocardiographic findings and multidisciplinary specialty team follow‐up during pregnancy. Patient consent was obtained.

## 2. Case Presentation

A 30‐year‐old, Hispanic pregnant G3P2002 woman with dichorionic/diamniotic twin gestation at 37 weeks 0/7 days presented in obstetrics triage with chief complaints of severe right‐sided chest pain with radiation to her back, nausea, and vomiting. Her symptoms began the morning of the presentation.

Her medical history is complicated by MFS with ocular manifestation, atrial septal defect (repaired with an Amplatzer device), mitral valve prolapse, GERD, and glaucoma. She was diagnosed with MFS in 2016 based on the Revised Ghent Nosology. There was no family history of MFS, cardiac disease, or sudden death. She had prior uncomplicated term vaginal deliveries (in 2017 and 2010). Her social history was negative for tobacco or alcohol use.

An echocardiogram was performed at the second trimester and showed a normal‐sized aortic root diameter (3.3 cm), a grossly normal aortic arch (not well seen), an ejection fraction of 58%, and a well‐seated atrium septal defect closure device in place in the interatrial septum. This exam was unchanged from the initial studies. The abdominal aorta was not imaged at that initial assessment.

Her blood pressure remained within normal limits throughout pregnancy, with systolic pressures of 120–139 mm Hg and diastolic pressures of 59‐74 mm Hg. She was not on any blood pressure‐controlling medication during the pregnancy. She had a repeat echocardiogram performed at 33 6/7 weeks with no clinical concerns, and no additional follow‐up or images were recommended before delivery.

Per cardiology consultation, the patient′s WHO risk was Class II–III with class III intermediate increased risk of maternal mortality or moderate to severe increase in morbidity, 11%–19% maternal cardiac event rate. It was recommended that she follow up with cardiology once per trimester. She was cleared by cardiology for vaginal delivery anticipated at 38 0/7–38 6/7 weeks of gestation.

Her admission physical exam was unremarkable, except for excruciating pain. She was also found to be in early labor with a cervical dilation of 5 cm. The fetal heart rates of both babies were reassuring. Vital signs were stable: blood pressure, 137/81 mm Hg; pulse, 75; temperature, 36.9°C; respiratory rate, 16; SpO2, 100%. Secondary to high clinical suspicion of aortic dissection, the patient was urgently taken to a CT angiogram with/without contrast. EKG was normal. Labetalol was initiated with the goal of maintaining blood pressure below 120 mm Hg, and avoidance of Valsalva was recommended if delivery was anticipated.

**Figure 1:**
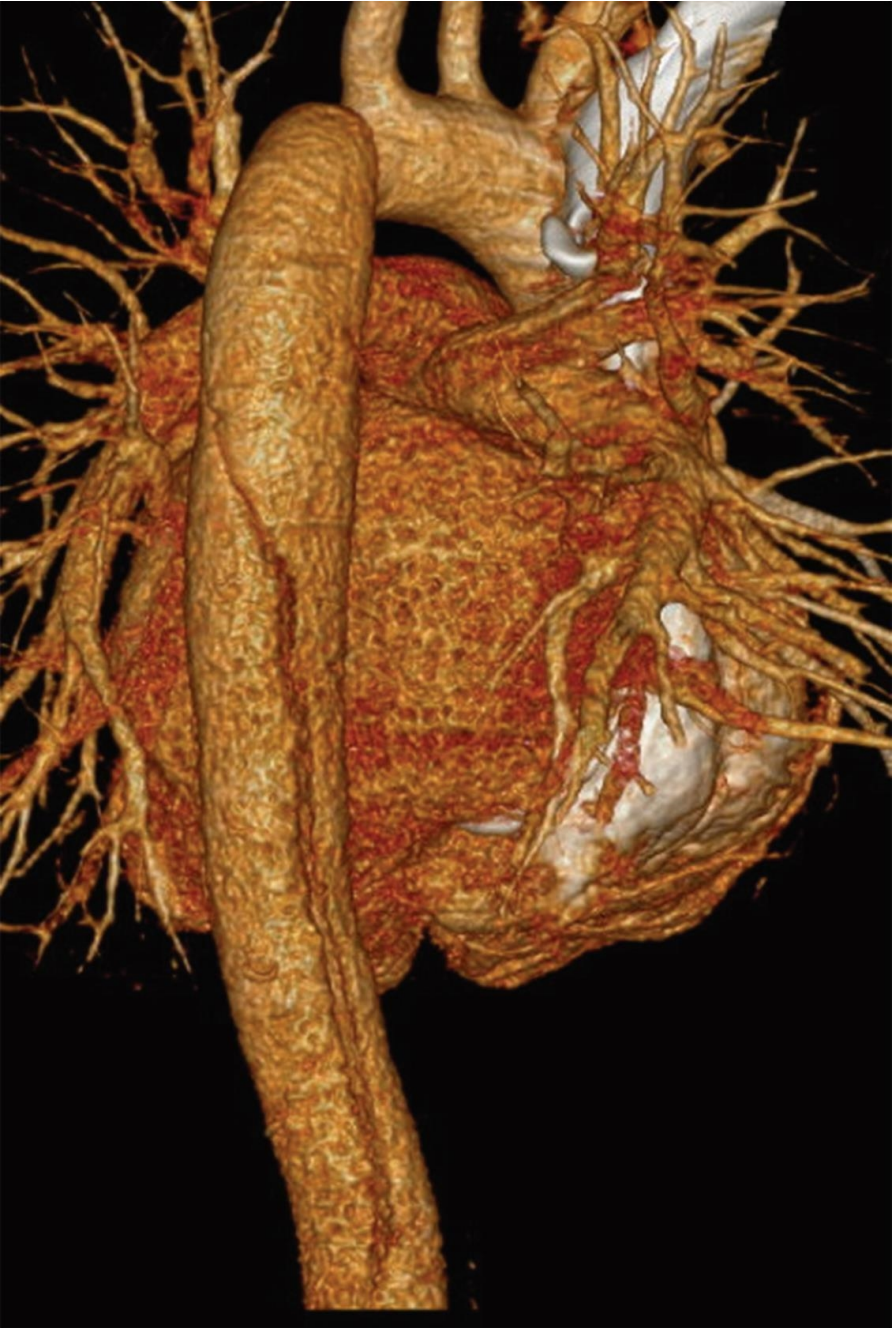
CT angiogram chest with and without contrast.

**Figure 2:**
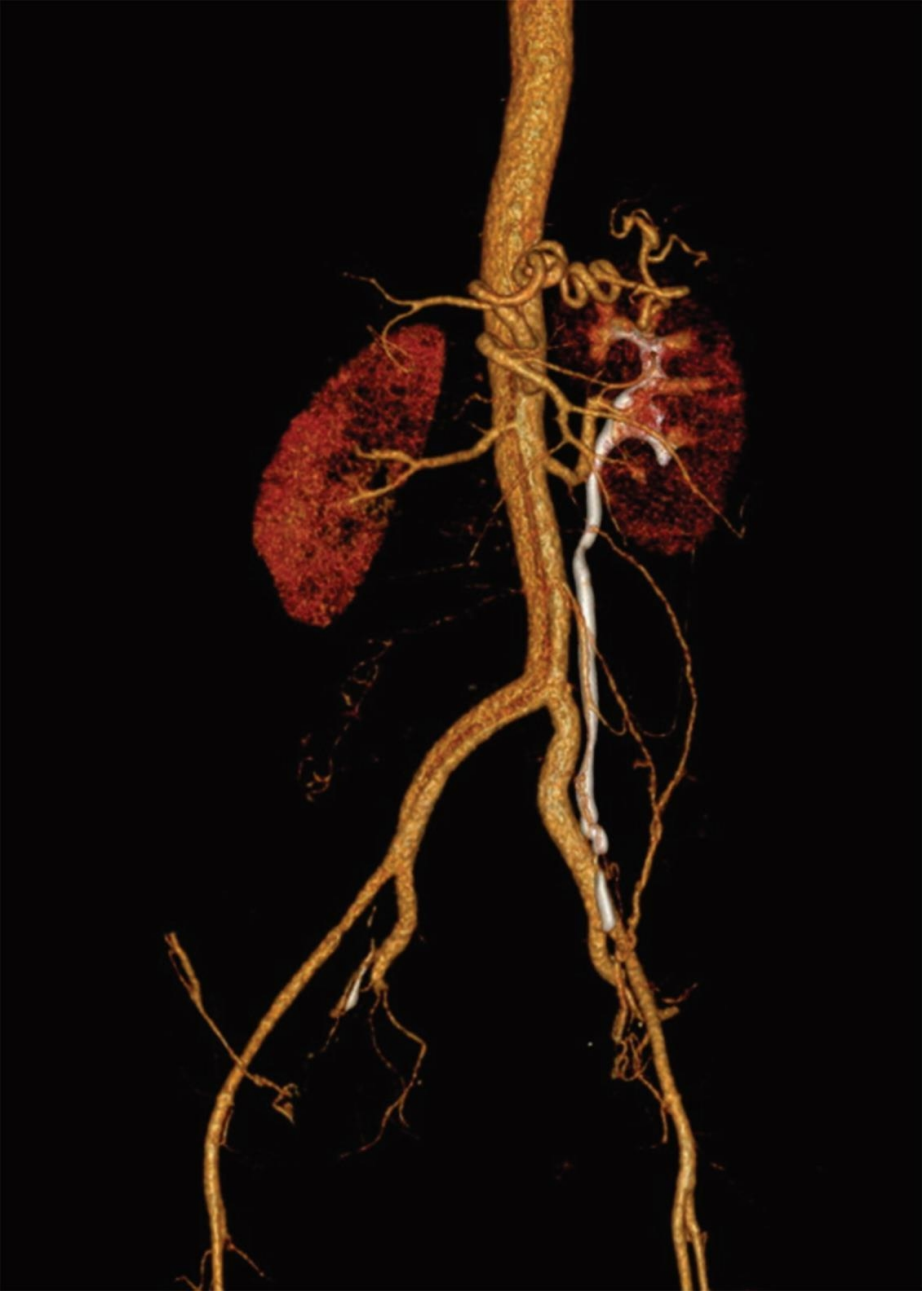
CT angiogram of abdomen/pelvis with and without contrast.

CT chest/abdomen with angiogram showed Stanford Type B aortic dissection extending from just distal to the left subclavian artery extending through the abdominal aorta into the right common iliac artery. The true, posterior, and left lateral lumens give rise to the celiac axis, the superior mesenteric artery (SMA), the left renal artery, and the inferior mesenteric artery (IMA). The right renal artery arises from the false lumen. There is no mediastinal hematoma or aneurysmal dilatation of the aorta. The uterus is gravid with twin pregnancy. CT images reflected in Figures 1 and 2.

Due to imminent delivery and aortic dissection, the patient was emergently taken into the OR for a cesarean section. All teams, including vascular, anesthesia, CT surgery, and interventional radiology, were present in the OR during the cesarean section. The Cesarean section was uncomplicated, with a quantitative blood loss of 704 cc. The twins were delivered weighing 2390 and 2060 g and having Apgar scores of 2/3 and 2/5, respectively.

After delivery, the patient was admitted to the ICU for postoperative care, BP control pressure, and conservative management of Type B dissection. Her postoperative course was uncomplicated overall. The patient decided to leave the hospital against medical advice on postoperative Day 5.

The patient was scheduled for CT images and cardiology follow‐up within a month of discharge.

## 3. Conclusion

We describe a case of Stanford Type B aortic dissection in the third trimester with MFS and twin gestation with initial aortic root < 45 mm. In this case, timely diagnosis and prompt intervention by a multidisciplinary team led to favorable maternal and fetal outcomes.

This pregnant woman received sufficient prenatal care, with an overall uneventful prenatal course, until late in the third trimester. Because MFS is classically associated with Type A dissection, the focus has been on measuring the aortic root in predicting the risk of complications. In this case, the thoracic and abdominal aorta are challenging to visualize with transthoracic echo and were not visualized during prenatal cardiac evaluation. However, there is insufficient evidence to conclude whether aortic root dilation also predicts Type B dissection. Upon reviewing the literature, there is no clear association between aortic diameter and the type of dissection. Minsart et al. prospectively followed 21 pregnancies in women with MFS and reported two complications, both of which were Type B dissections in women with an aortic root < 40 mm [8]. High‐risk pregnancy specialists and cardiologists should always keep in mind that small numbers of MFS pregnancy‐related complications will occur more distally along the aorta. Therefore, the entire aorta should be imaged before or early in pregnancy [9, 10]. There is no established cutoff and location of measurements or standardized frequency of serial descending and abdominal aorta measurements to predict a patient′s risk for aortic dissection. However, it may be considered in patients with an aneurysmal dilation of distal portions of the aorta at baseline. Whole aorta imaging using CT may be warranted, given that Type B dissections may not be visualized by the routine transthoracic echocardiographic assessment of the aortic root.

## Funding

No funding was received for this manuscript.

## Conflicts of Interest

The authors declare no conflicts of interest.

## Data Availability

The data that support the findings of this study are available on request from the corresponding author. The data are not publicly available due to privacy or ethical restrictions.
